# Postoperative Surprises: A Carcinoid Tumor Within a Perforated Meckel's Diverticulum

**DOI:** 10.7759/cureus.63834

**Published:** 2024-07-04

**Authors:** Tara Ranjbar, Jeffrey Robles, Dillon Rogando, Adham Ahmed, Sourodip Mukharjee, Erika Clarke, Crystal Antony, Kalena H Liu, Dhruv Patel, Indraneil Mukherjee

**Affiliations:** 1 General Surgery, Staten Island University Hospital, New York, USA; 2 General Surgery, City University of New York (CUNY) School of Medicine, New York, USA; 3 Minimally Invasive Surgery, Staten Island University Hospital, New York, USA

**Keywords:** meckel's diverticulum, general surgery and colorectal surgery, screening colonoscopy, colonoscopy, sigmoidectomy, carcinoid tumour

## Abstract

Meckel's diverticulum, a true diverticulum originating from the incomplete closure of the vitelline duct during embryologic development, rarely presents with carcinoid tumors. The coexistence of a Meckel's diverticulum and carcinoid tumor following laparoscopic sigmoid colectomy for diverticulitis is an uncommon phenomenon, with limited documented cases in the literature.

We present a case of a 74-year-old male with a past medical history of hypertension and diverticulitis who underwent a laparoscopic sigmoid colectomy for dysplastic and cancerous changes of a polyp revealed during a screening colonoscopy. Initially, the patient’s postoperative journey was uneventful with the resumption of regular bowel movements and favorable diet progression. However, he later presented to the emergency department for worsening abdominal pain and distension. Imaging prompted surgical intervention due to perforation and obstruction, resulting in the identification of a carcinoid tumor within a perforated Meckel's diverticulum.

This case highlights the intricate challenges of postoperative complications, particularly the unexpected emergence of Meckel’s diverticulum pathology following a colectomy. The atypical presentation, featuring a carcinoid tumor within a perforated Meckel’s diverticulum, underscores the importance of evaluating abdominal symptoms postoperatively.

## Introduction

A Meckel's diverticulum, arising from the incomplete closure of the vitelline duct during embryologic development, represents a true diverticulum averaging 3 cm in length, typically found within 100 cm of the ileocecal valve [1]. While its lining often comprises intestinal mucosa, it may also contain heterotopic tissues, such as gastric mucosa and ectopic pancreas [2]. Although the majority of Meckel's diverticula remain asymptomatic, approximately 2% of the population experiences complications. In the pediatric population, 50% of cases are seen before the age of two meanwhile in the adult population, most complications occur between the ages of 30-40 years old. Symptoms associated with a Meckel's diverticulum range from lower gastrointestinal bleeding to inflammation, intestinal obstruction, intussusception, and, more rarely, neoplastic transformation [3].

Carcinoid tumors, slow-growing neoplasms originating from neuroendocrine cells capable of secreting bioactive amines, are predominantly found in the gastrointestinal tract (60%) and the tracheobronchial tree (25%). Occurrences in the ovaries, testicles, and kidneys are rare [4]. Symptomatic manifestation, known as carcinoid syndrome, typically occurs with metastatic disease to the liver and presents with episodic flushing (84%), watery diarrhea (70%), and heart disease (37%) [5].

The concurrent presence of Meckel's diverticulum and carcinoid tumors is infrequent, accounting for 0.5-3.2% of Meckel-related complications involving malignancy [3]. In this report, we present a unique case involving a carcinoid tumor within a perforated Meckel's diverticulum, an occurrence after a laparoscopic sigmoid colectomy performed for dysplastic polyps.

## Case presentation

We present a case involving a 74-year-old male with a medical history of hypertension and a single episode of diverticulitis. Having undergone a screening colonoscopy that revealed three tubulovillous adenoma sigmoid polyps, the patient was scheduled for a laparoscopic sigmoid colectomy. One of the tubulovillous adenomas showed high-grade dysplastic changes with negative margins free of adenomatous changes with the others showing focal intra-mucosal carcinoma with positive margins for adenomatous changes. During the surgical procedure, significant sigmoid adhesions were encountered, however, the endoscopic tattoo was identified and sigmoid colon resection was performed with intracorporeal primary anastomosis with the circular stapler. During the insertion of the anvil into the proximal limb, a wound protector was used. Following surgery, the patient's recovery progressed as follows: clear liquid diet initiation on postoperative day (POD) 1, transition to a regular diet on day 3 with regular bowel movements, and monitoring for abdominal distension for an additional two days. On POD 5, the patient was comfortable, continued having regular bowel movements, and consumed over 70% of the diet, and was able to be discharged that day.

The patient later presented to the emergency department due to worsening abdominal pain and distention on POD 8. He denied nausea or vomiting and reported regular bowel movements except for the previous day but was still passing flatus. Initial vital signs included a blood pressure of 144/77 mmHg, a pulse of 114 beats/min (reducing to 88 beats per minute while on lactated ringers at a rate of 120 mL/hour), a respiratory rate of 20 breaths/min, a temperature of 96.5°F/35.8°C, and a pain level of 5/10. Despite appearing awake and alert, the patient displayed mild distress with significant abdominal distention. On examination, the abdomen was soft with diffuse mild tenderness without guarding or rebound tenderness.

Diagnostic evaluation included imaging and laboratory results revealing notable values such as a white blood cell count of 13.6 x 10^9/L, hemoglobin of 14.1 gm/dL, hematocrit of 41.1%, platelet count of 241,000/mL, glucose of 110 mg/dL, blood urea nitrogen of 17 mg/dL, serum creatinine of 0.98 mg/dL, and albumin of 3 gm/dL.

**Figure 1:**
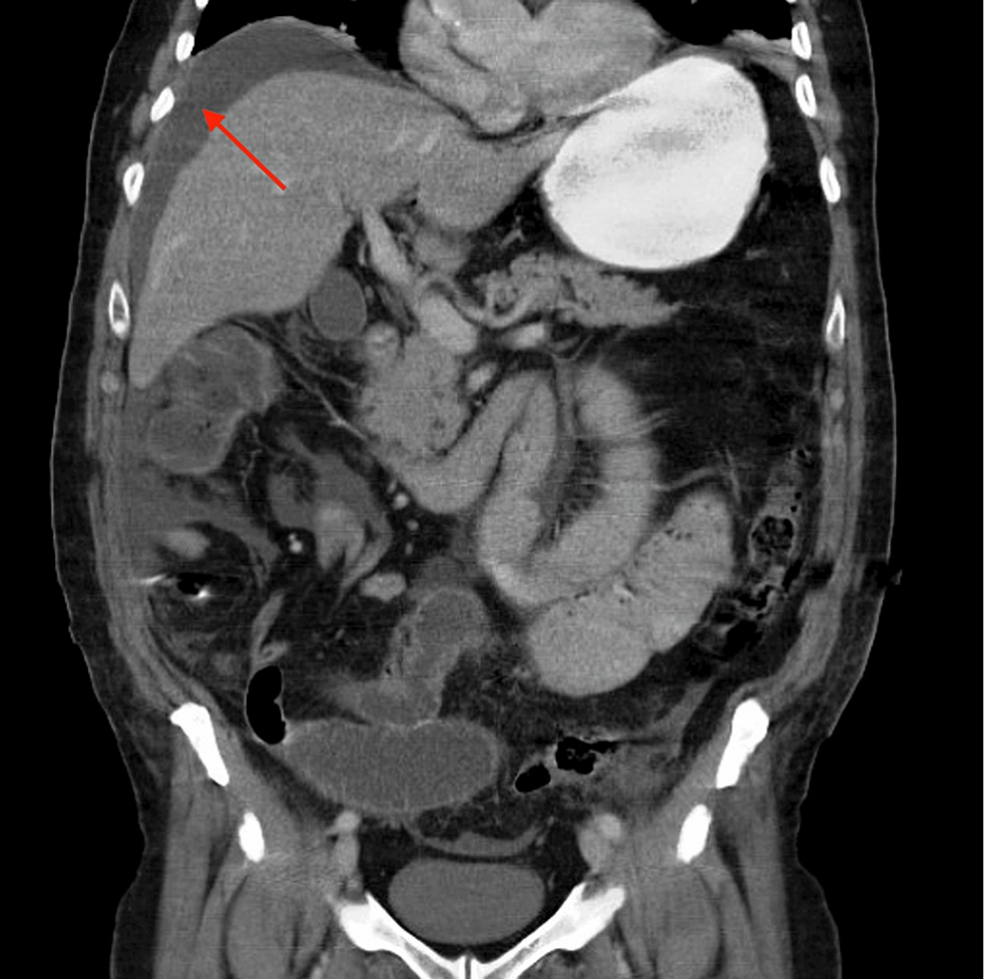
CT Abdomen and Pelvis Free intraperitoneal collection located between the liver and the diaphragm.

**Figure 2:**
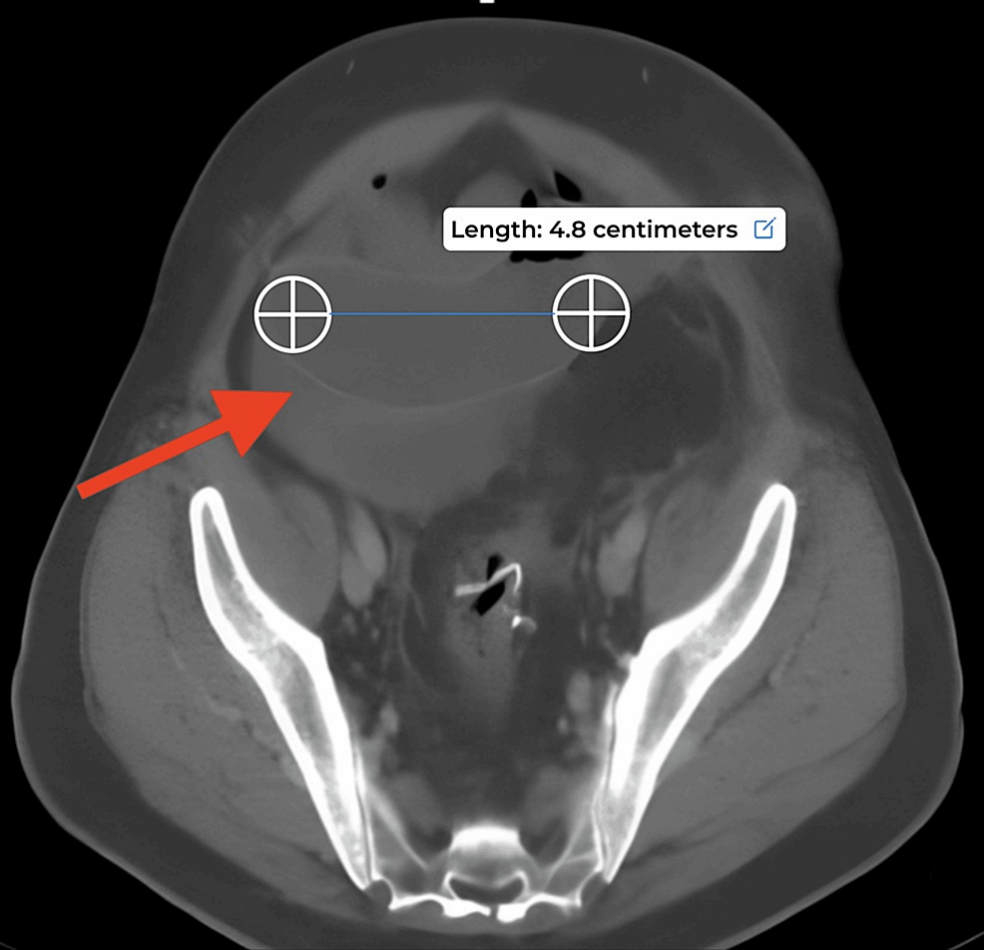
CT Abdomen and Pelvis Multiple dilated loops of the small bowel, measuring up to 4.8 cm, with an apparent transition point in the mid-abdomen.

Given the clinical stability, the patient underwent an abdominal CT scan with IV contrast (Figures 1-2) which showed an intraperitoneal collection, predominately in the upper abdomen between the liver and the diaphragm along with multiple dilated loops of small bowel up to 4.8 cm in length with a transition point. Upon diagnosis with a small bowel obstruction or possible post-surgical ileus, a nasogastric tube and urinary catheter were inserted, and the patient was prepared and consented to surgery. Initially performed as a diagnostic laparoscopy, the procedure revealed a copious amount of murky, bile-stained, non-foul-smelling fluid upon trocar introduction. Despite extensive adhesiolysis, the source of perforation and obstruction could not be identified, prompting a conversion to a laparotomy. The examination of the rectal anastomosis, large bowel, and appendix revealed normal findings. During the exploration of the small bowel, a portion adhered to the lower right abdomen was bluntly dissected, revealing a small outpouching resembling Meckel's diverticulum 1.3 cm long and 100 cm from the ileocecal valve, which had perforated at its tip. The segment was resected and a 28 EEA circular stapler (Medtronic, Dublin, Ireland) was used for a side-to-side re-anastomosis, followed by an abdominal washout. The fascia and skin were closed, and the patient, requiring postoperative intensive care due to hemodynamic instability requiring pressors, experienced a successful recovery, ultimately being discharged on POD 12. Pathology results indicated a 12 cm x 3.1 cm rectosigmoid colon excised with scattered diverticula and no microscopic evidence of malignancy in the colon or in the 10 lymph nodes collected. Within the perforated Meckel's diverticulum, a well-differentiated carcinoid tumor measuring 2 x 1.2 cm was identified involving the serosal surface. The tumor's stage was found to be pTa4a, pN0, pMX, and the tumor immunohistochemical staining was positive for chromogranin, synaptophysin, pan-cytokeratin, and D2-40 all of which support the diagnosis of carcinoid tumor. The proximal and distal surgical margins of resection were free of tumor.

## Discussion

The presented case of a carcinoid tumor within a Meckel's diverticulum, discovered incidentally following a laparoscopic sigmoid colectomy for diverticulitis, underscores the complexity of gastrointestinal pathologies and highlights the importance of thorough investigations in postoperative complications. Carcinoid tumors exhibit a broad distribution, with a prevalent occurrence in the gastrointestinal tract, specifically the small intestine (60%), of which 42% are in the small intestine [6]. Primary malignancies of Meckel's diverticulum are very rare with a reported incidence of 0.5-3.2% [7]. In the study by Modlin et al., it was found that the incidence of carcinoid tumors located in the Meckel's diverticulum was reported at 0.74%. However, a notable finding emerged: despite its small size, Meckel’s diverticulum exhibited the highest rate of carcinoid transformation per square centimeter of mucosal surface.

In a 1988 review of the literature, there were only 52 reported cases of carcinoid tumor in Meckel’s diverticulum [8]; a more recent study identified 121 reported cases [7]. The extreme rarity of this lesion makes the clinical behavior and outcome somewhat difficult to predict. Statistical evidence indicates that solitary carcinoid tumors smaller than 1 cm infrequently metastasize and are generally surgically curable, while larger tumors or those exceeding 2 cm metastasize in almost 90% of cases [9]. Carcinoids localized in the appendix and colorectum typically follow a more benign course, while those in the small bowel and bronchi exhibit a more aggressive clinical trajectory [10].

A very possible differential diagnosis of such a large amount of intraperitoneal collection could only mean an overt perforation, however, it was not very evident in this case. This advanced T4 tumor had definitely invaded through all the layers of the Meckel's diverticulum, but must have happened sub-acutely. The patient was definitely symptomatic from the carcinoid even before the sigmoid colectomy. The stress of the surgery might have precipitated matters in an unknown way.

Our case is an unusual manifestation, emphasizing the need for individualized approaches to diagnosis and management. The postoperative trajectory of our case underscores the pivotal role of multidisciplinary collaboration in handling intricate gastrointestinal diseases and the potential emergence of secondary pathologies. Our patient will be monitored by measuring urinary 5-hydroxyindoleacetic acid (5-HIAA) levels every three to six months to ensure peritoneal disease does not exist after the tumor is found within the perforation.

## Conclusions

Our reported case involves a 74-year-old male with a history of hypertension and a single episode of diverticulitis who underwent a laparoscopic sigmoid colectomy due to dysplastic tubulovillous adenomas identified during a screening colonoscopy. Postoperatively, the patient exhibited an uneventful recovery, with the resumption of regular bowel movements and tolerance of a regular diet. However, the patient's clinical course took an unexpected turn when he returned to the emergency department with worsening abdominal pain and distention. Despite regular bowel movements, imaging revealed a large collection of intraperitoneal fluid and small bowel dilatation, necessitating urgent surgical intervention. Diagnostic laparoscopy converted to laparotomy revealed a carcinoid tumor within Meckel's diverticulum that had perforated at its tip. The diverticulum was resected, and the patient underwent an abdominal washout. Although postoperative intensive care was required for pressors, the patient did well and was discharged on POD 12.

This case demonstrates the challenges associated with postoperative complications and the unanticipated discovery of Meckel's diverticulum after a colectomy. The atypical presentation of a perforated Meckel's diverticulum further signifies the importance of recognizing high-risk abdominal symptoms, especially in patients with a history of diverticulitis and prior abdominal surgeries. This case contributes valuable insights into the complexities of gastrointestinal pathology and postoperative care, prompting further research to refine diagnostic and management strategies in similar clinical scenarios, such as possible cytoreductive surgery (CRS)/hyperthermic intraperitoneal chemotherapy (HIPEC) therapy for the perforation as long as there are no signs of sepsis present.
